# The Development of an Isotope Dilution Mass Spectrometry Method for Interleukin-6 Quantification

**DOI:** 10.3390/ijms25126777

**Published:** 2024-06-20

**Authors:** Zetao Yu, Jing Wang, Wenqiang Xia, Yuemin Wang, Yafen Zhang, Jintian Tang, Haifeng Cui, Xiaoying Yang, Chenchen Bao, Zihong Ye

**Affiliations:** 1 Zhejiang Provincial Key Laboratory of Biometrology and Inspection & Quarantine, College of Life Sciences, China Jiliang University, Hangzhou 310018, China; yuzetaoyzt@163.com (Z.Y.); z240845304@163.com (J.W.); wym@cjlu.edu.cn (Y.W.); yfzhang@cjlu.edu.cn (Y.Z.); jintiantang@cjlu.edu.cn (J.T.); hfcui@cjlu.edu.cn (H.C.); 18206176811@163.com (X.Y.); m13736388872_1@163.com (C.B.); 2 Institute of Crop Science, College of Agriculture and Biotechnology, Zhejiang University, Hangzhou 310012, China; wqxia.zj@zju.edu.cn

**Keywords:** interleukin-6, signature peptides, isotope dilution mass spectrometry (IDMS)

## Abstract

Inflammatory responses and tumor developments are closely related, with interleukin-6 (IL-6) playing important roles in both processes. IL-6 has been extensively identified as a potential tumor biomarker. This study developed an isotope dilution mass spectrometry (IDMS) method for quantifying IL-6 based on signature peptides. These peptides were screened by excluding those with missed cleavage or post-translational modification. The method’s accuracy was verified using amino acid-based IDMS, in which purified IL-6 protein samples were quantified after hydrolyzing them into amino acids, and no significant difference was observed (*p*-value < 0.05). The method demonstrated good linearity and sensitivity upon testing. The specificity and matrix effect of the method were verified, and a precision study showed that the coefficient of variation was less than 5% for both the intra-day and inter-day tests. Compared to immunoassays, this method offers distinct advantages, such as the facilitation of multi-target analysis. Furthermore, the peptides used in this study are much more convenient for storage and operation than the antibodies or purified proteins typically used in immunoassays.

## 1. Introduction

Interleukin-6 (IL-6) is a typical multifunctional cytokine involved in immunomodulation, hematopoiesis, and inflammatory processes [1]. It belongs to the IL-6 family, which includes IL-6, IL-11, IL-27, ciliary neurotrophic factor (CNTF), cardiotrophin 1 (CT-1), cardiotrophin-like cytokine (CLC), oncostatin M (OSM), and leukemia inhibitory factor (LIF). Members of this family possess the dual properties of promoting inflammation and inhibiting its progression [2]. IL-6 family members have been identified as biomarkers for clinical testing through the screening of inflammatory mediators in various fluids, including saliva, serum, and bronchoalveolar lavage fluid [3]. IL-6 levels are significantly up-regulated in response to organismal infections, making it a widely used biomarker for clinical inflammation testing [4]. Additionally, IL-6 plays diverse physiological roles in organisms. It acts as a maturation agent for B lymphocytes [5], stimulating the synthesis and secretion of various immunoglobulins [6]. IL-6 also induces the proliferation and secretion of thymic T cells and peripheral T cells [7]. Furthermore, IL-6 is an inducer of the C-reaction protein [8]. The blockade of IL-6 signaling by anti-IL-6 autoantibodies inhibits serum CRP elevation [9].

IL-6 plays a crucial role in the tumor microenvironment [10], and it is the most abundant cytokine in this setting. Research results indicate that IL-6 is highly expressed in almost all types of tumors. For instance, in a study involving 55 patients diagnosed with severe lung cancer, elevated IL-6 levels were found to be significantly associated with poorer performance status and disease progression. Patients with high IL-6 levels exhibited worse Eastern Cooperative Oncology Group scores and were more likely to experience disease progression [11]. The results of recent clinical studies have additionally shown that IL-6 levels are associated with clinical stage, poor prognosis, and survival in patients with various types of cancer, including cervical cancer [12], breast cancer [13], ovarian cancer [14], prostate cancer [15], and colorectal cancer [16].

The accurate quantification of IL-6 has become necessary for the application of IL-6 in clinical practice. Currently, researchers classify protein quantification techniques into two main groups: immunoassays and mass spectrometry [17]. Immunoassays (e.g., ELISA) quantify proteins by measuring the complexes formed through protein–antibody interactions, which are commonly used in clinical testing [18,19]. However, immunoassays are limited to measuring free proteins in solution, which can introduce errors in test results. Post-translational modifications of proteins and the effects of autoantibodies can impact the clinical test results of immunoassays, leading to differences in results between different testing platforms [20]. Isotope dilution mass spectrometry (IDMS) is a highly sensitive and accurate detection method that is traceable to the International System of Units (SI) [14,15]. By using specific isotopic markers as internal standards, it can reduce the impact of interfering compounds on quantitative data and improve reproducibility. IDMS-based protein quantification methods involve the use of a bottom-up approach to hydrolyze denatured proteins into amino acids or signature peptides. Isotope-labeled amino acids or signature peptides are used as internal standards. As the amino acids in different proteins are similar, amino acid analysis is commonly employed for the quantification of purified proteins but lacks specificity. In contrast, signature peptides are specific to proteins, as they are produced via the enzymatic digestion of proteins. Specifically, trypsin selectively hydrolyzes peptide chains at the carboxyl end of lysine (K) and arginine (R), generating some specific peptides, which can be used for the protein quantification. IDMS based on signature peptides allows the high-throughput data acquisition of hundreds to thousands of targets under optimized instrumental acquisition modes through the multiple reaction monitoring of triple quadrupole mass spectrometry [21].

At present, IDMS based on signature peptides has gradually become a standardized reference method for quantifying protein concentration. The purpose of the following paper is to develop and validate an accurate method for IL-6 quantification based on IDMS by screening signature peptides and method optimization, providing a research basis for IL-6 protein quantification and clinical testing.

## 2. Results and Discussion

### 2.1. Quantitative Method Establishment

#### 2.1.1. Signature Peptide Selection

Primary screening was conducted based on the sequence characteristics. Trypsin cleaved the IL-6 protein on the C terminus of lysine and arginine to produce a series of peptides. However, if any site is not cleaved, the predicted peptides may not be formed or may diminish, leading to erroneous protein quantification results. To identify peptides with missed cleave sites, trypsin-hydrolyzed products of IL-6 were analyzed via high-resolution mass spectrometry (Table 1). The results revealed that the numbered arginine or lysine sites of DVAAPHR^44^, K^69^, MAEK^98^, and LQAQNQWLQDMTTLILR^196^ were not fully cleaved.

In contrast, glycosylation changes the molecular weight and charge state of peptides. This process will result in differences in the properties between the peptides resulting from trypsin digestion and the manually synthesized peptide, leading to inaccurate protein quantification. Based on the report and prediction results, it was found that numbered serine or threonine sites in the MNSFSTSAFGPVAFSLGLLLVLPAAFPAPVPPGEDS^36^K, QPLT^48^S^49^S^50^ER, ETCN^73^K, and NLDAIT^165^T^166^PDPT^170^T^171^NAS^174^LLTK peptides may undergo glycosylation.

**Figure 1:**
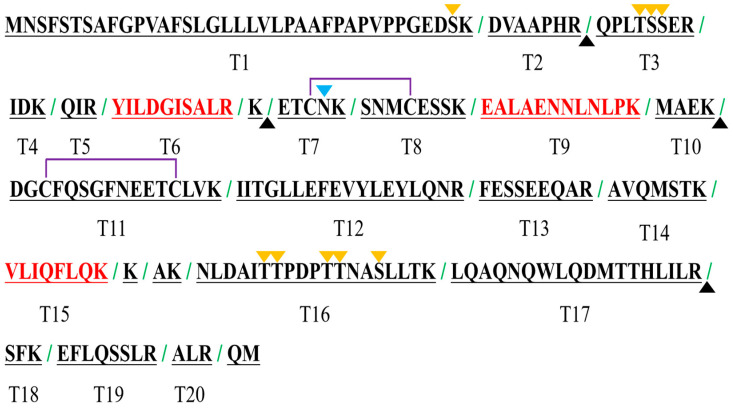
Summary of sequence characteristics. Expected trypsin cleavage sites are indicated by green slashes; missed cleavage sites are indicated by black triangles; the reported glycosylation site is indicated by a blue triangle; predicted glycosylation sites are indicated by yellow triangles; reported disulfide bonds are indicated by the purple connecting lines; and signature peptides for IL-6 quantification used in this study are marked by red highlights.

**Figure 2:**
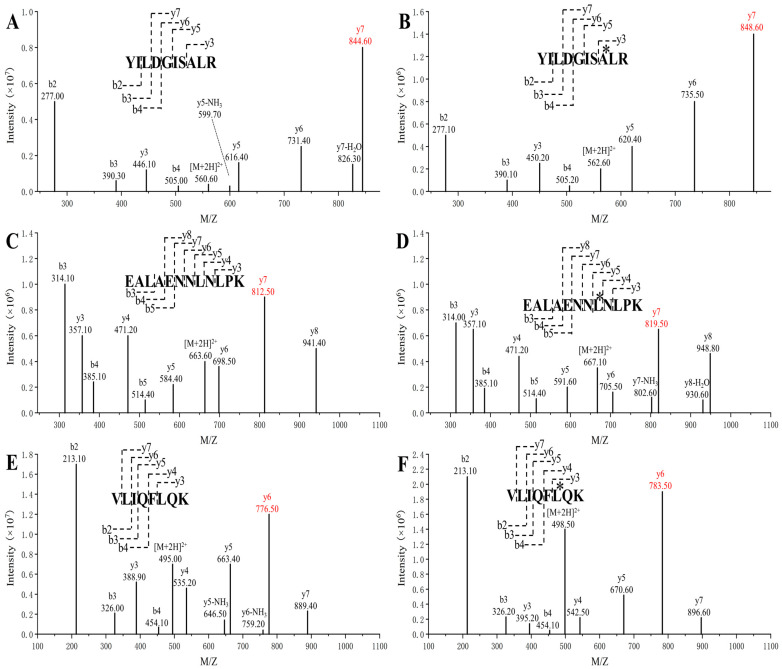
MS2 spectra of T6, T9, and T15 and their isotope-labeled peptides: (**A**) T6 (YILDGISALR); (**B**) T6* (YILDGISA(^13^C_9_,^15^N)LR); (**C**) T9 (EALAENNLNLPK); (**D**) T9* (EALAENNL(^13^C_6_,^15^N)NLPK); (**E**) T15 (VLIQFLQK); (**F**) T15* (VLIQFL(^13^C_9_,^15^N)QK). Amino acids marked with * indicate the isotopic labeling. The fragment ions used for detection are highlighted by red text.

Missed cleavage sites, probable glycosylation sites, and disulfide bonds are shown in Figure 1, and related peptides are excluded for IL-6 protein quantification. Peptides that were located at the termini were also excluded because they were subject to hydrolysis. Finally, signature peptides T6 (YILDGISALR), T9 (EALAENNLNLPK), and T15 (VLIQFLQK) were selected for IL-6 quantification. These peptides were manufactured via solid-phase peptide synthesis to create an in-house reference material. The purity of the synthesized T6, T9, and T15 peptides was 95.62%, 97.19%, and 98.89%, respectively, according to the HPLC analysis results. The isotope-labeled peptides, T6* (YILDGISA(^13^C_9_,^15^N)LR), T9* (EALAENNL(^13^C_6_,^15^N)NLPK), and T15* (VLIQFL(^13^C_9_,^15^N)QK), were also synthesized to act as internal references. All of the unlabeled and isotope-labeled peptides were confirmed using MS2 spectra (Figure 2). The precursor ions and fragment ions used for analysis are indicated in the figure, and the high-intensity fragment ions were chosen.

#### 2.1.2. Mass Fraction of the Signature Peptides in In-House Reference Materials

During the solid-phase synthesis of signature peptides, impurities such as moisture and trifluoroacetic acid can be introduced. This means that the content of peptides in the stock solutions should be calculated based on both the weight of the in-house reference materials, and the mass fraction of the signature peptides in them. The signature peptide was hydrolyzed into amino acids and then quantified via IDMS. Retention times for the detection of amino acids are shown in Figure S1. Certified reference materials of amino acids were used for quantification. The mass fraction of each signature peptide in the in-house reference material was calculated based on amino acid content and peptide purity. The mass fractions of the signature peptides T6, T9, and T15 in the in-house reference materials are shown in Table 2. For T9 and T15, the results of the statistical analysis suggest no significant difference between the quantification results of the two amino acids (Student’s *t*-test, *p*-value > 0.05).

#### 2.1.3. Optimization of Trypsin Digestion Time

**Figure 3:**
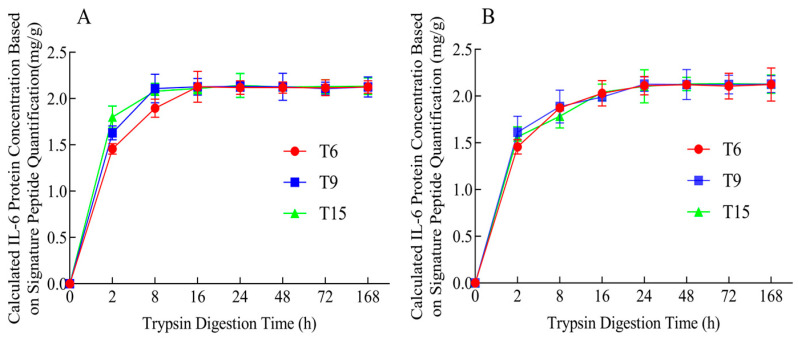
(**A**) the calculated concentration of IL-6 diluted in buffer (20 mM Tris pH 8.0, 150 mM NaCl); (**B**) the calculated concentration of IL-6 diluted in human serum. The samples were trypsin-hydrolyzed for different incubation time and detected via signature peptide-based IDMS. Three replications were conducted for each time point.

The complete digestion of protein is crucial for accurate quantification. IL-6 was hydrolyzed for 2, 8, 16, 24, 48, 72, and 168 h, following the process described in Section 3.5 (Figure 3A). In-house reference materials of signature peptides were used as a calibrator. The IL-6 protein samples were rapidly digested by trypsin within 0 to 16 h. The rate of digestion slowed after 24 h and reached a maximum at 48 h. To ensure the complete enzymatic digestion of the IL-6 protein, the trypsin digestion time for the IL-6 samples was set at 48 h.

Additionally, we compared the enzymatic digestion rates of IL-6 when diluted in human serum versus buffer (20 mM Tris pH 8.0; 150 mM NaCl). Following the sample preparation method outlined in Section 3.5, the IL-6 samples diluted in human serum were digested for 2, 8, 16, 24, 48, 72, and 168 h, with instrument detection performed as outlined in Section 3.6. The results, based on three signature peptides (Figure 3B), indicated that IL-6 was also completely digested within 48 h. However, the digestion rate was slightly lower than when diluted in buffer.

### 2.2. Method Validation

#### 2.2.1. Linearity and Sensitivity

**Figure 4:**
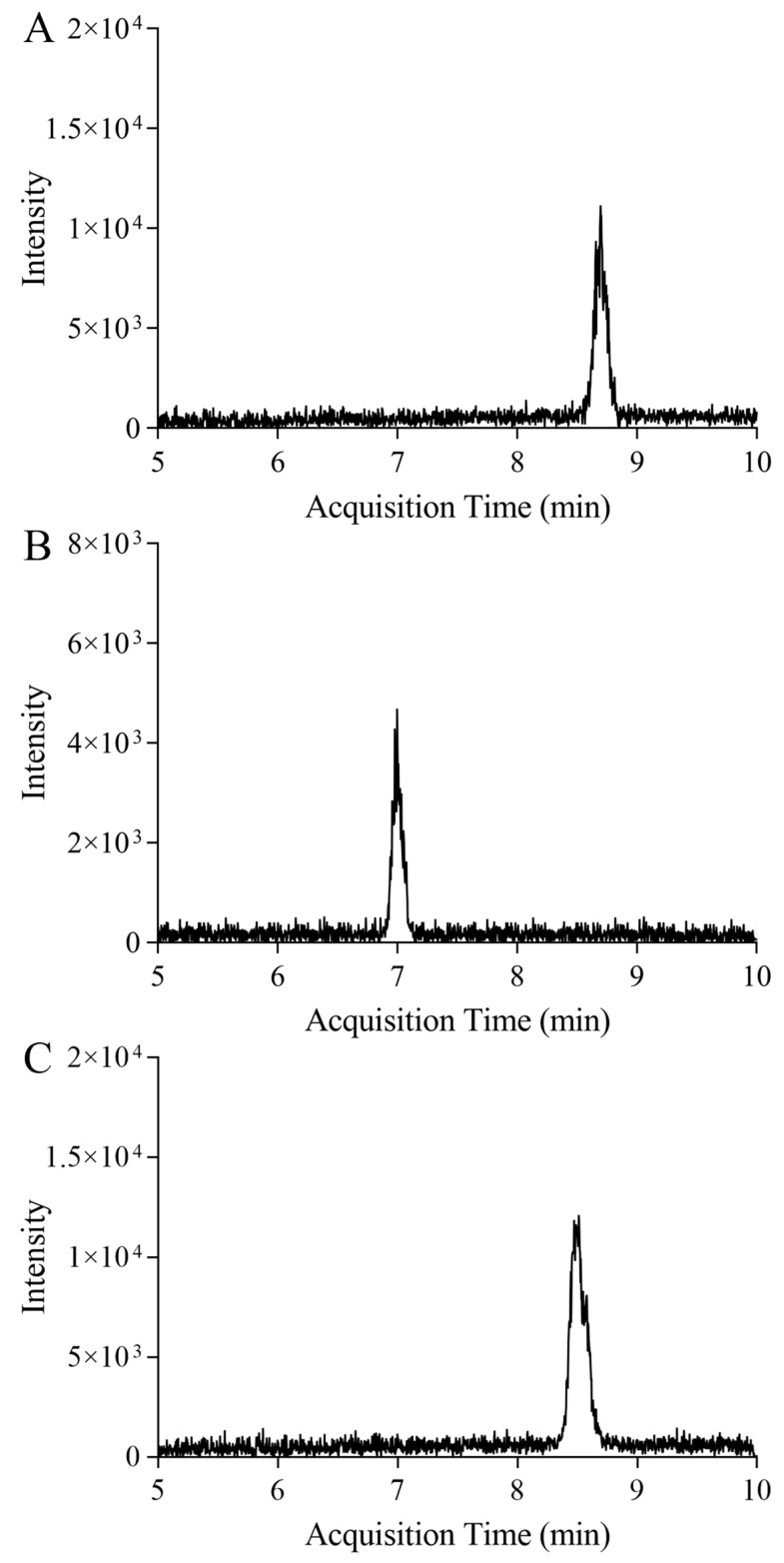
Chromatograms of signature peptides at the low limit of quantification level. (**A**–**C**) represent the chromatograms of T6, T9, and T15, respectively.

For the calibrators, the in-house reference materials of the signature peptides were diluted into a series of concentrations using ultrapure water (5000, 2000, 1000, 500, 200, 100, 50, 20, 10, 5, 2, 1, 0.5, 0.2, and 0.1 ng/g), and the isotope-labeled internal reference was added to obtain a final concentration of around 100 ng/g. The low limit of detection is for concentrations with a signal-to-noise ratio of 3:1, and the low limit of quantification is for concentrations with a signal-to-noise ratio of 10:1. The chromatograms of the low limit of quantification of the signature peptides are shown in Figure 4. The results for linearity, low limit of detection, and low limit of quantification for each signature peptide are shown in Table 3. The correlation coefficients for the calibration curves of the T6, T9, and T15 peptides were 0.995, 0.999, and 0.995, respectively, indicating excellent linearity for the IDMS method based on these signature peptides.

#### 2.2.2. Accuracy

Purified recombinant IL-6 protein was first quantified via signature peptide-based IDMS (Table 4). One-way ANOVA was conducted to compare the quantification results of T6, T9, and T15. No significant difference was observed (*p*-value > 0.05).

The use of IDMS based on amino acids has been confirmed in several international metrological comparisons and is widely used in the quantification of reference materials. Purified recombinant IL-6 protein was also quantified via amino acid-based IDMS, with leucine, proline, and valine used for quantification. We analyzed the content of amino acids in the hydrolysis solution at different time points. After 36 h, the content of amino acids in the hydrolysis solution no longer increased, which indicated that the IL-6 protein was completely hydrolyzed (Figure S2). Therefore, the hydrolysis time was set to 36 h. The results of the IL-6 quantification based on the signature peptides and amino acid assay are shown in Table 5. The statistical analysis results suggested no significant difference (Student’s *t*-test, *p*-value > 0.05).

#### 2.2.3. Specificity

The signals of the ion pair of selected signature peptides were not detected when analyzing the tryptic hydrolysis products of the lysates of HEK293 cells, human serum, and bovine serum albumin. These results indicate that the specificity meets the criteria.

#### 2.2.4. Sample Matrix Effects

The purified IL-6 was added into the serum to prepare the samples of matrix effect detection. The detected IL-6 content in the samples was compared with the theoretical content, and the recovery rate was used to measure the matrix effects. Table 6 shows that the recovery rates of IL-6 in the high, middle, and low IL-6 concentration samples were 91.81%, 90.74%, and 86.36%, indicating that the matrix effects from the serum were limited.

#### 2.2.5. Precision

The intra-day and inter-day precision results are presented in Table 7. The precision of the method was evaluated as the coefficient of variation (CV). The data show that the intra-day and inter-day precision results for the samples of high, medium, and low concentrations are all less than 5%.

## 3. Materials and Methods

### 3.1. Reagents and Instruments

During the study, the following reagents and instruments were used: acetonitrile class “LC-MS” (Aladdin, Shanghai, China); formic acid, 98% pure, class “LC-MS” (JK Scientific, Beijing, China); lyophilized trypsin, sequencing grade (Promega, Madison, WI, USA); certified reference materials of leucine, proline, valine, and phenylalanine (National Institute of Metrology of China, Beijing, China); isotope-labeled amino acids of leucine, proline, valine, and phenylalanine (Cambridge Isotope Laboratories, Tewksbury, MA, USA); specific peptides (in-house reference materials) and isotope-labeled peptides (GL Biochem, Shanghai, China); and the Xevo TQD HPLC-MS/MS system (Waters, Milford, CT, USA).

Source of IL-6: Human IL-6 (NCBl accession: P05231.1) with a C-terminal His 8-tag was cloned into a pEZT-BM vector and expressed in HEK293F cells (Thermo Fisher Scientific, Milford, CT, USA) using the BacMam system (Thermo Fisher Scientific). The baculovirus was generated in Sf9 cells (Thermo Fisher Scientific) following the standard protocol and used to infect HEK293F cells at a ratio of 1:40 (virus:HEK293F, *v*/*v*) and supplemented with 10 mM sodium butyrate to enhance protein expression. The cells were cultured in suspension at 37 °C for 48 h and collected following centrifugation at 3000× *g* at 4 °C. Total protein was extracted using a mammalian protein extraction reagent (M-PER, Thermo Fisher Scientific) that included protease inhibitors (Thermo Fisher Scientific). The protein was purified via nickel affinity chromatography and eluted with a linear gradient of imidazole (20–500 mM). Afterwards, the eluents were loaded onto a PD-10 desalting column containing Sephadex G-25 (Cytiva, Marlborough, MA, USA) to remove excess imidazole, and the buffer was exchanged with 20 mM Tris pH 8.0, 150 mM. The IL-6 protein was further purified using size-exclusion chromatography HPLC (SEC-HPLC), resulting in a purity of over 99%.

### 3.2. Signature Peptide Selection

#### 3.2.1. Analysis of Missed Cleavage Sites

Based on the sequence of IL-6 searched from the UniProt protein database, the signature peptides produced by the trypsin treatment of IL-6 were predicted according to the characteristics of the trypsin selective cleavage of the arginine and lysine sites. These signature peptides were selected for the quantification of the IL-6 protein after screening.

Peptide fingerprinting is a commonly used method for protein identification. The sequence-specific peptides obtained during detection can be used to locate missed cleavage sites in trypsin digestion. The IL-6 treated with trypsin was separated via HPLC, followed by MS detection. The detected peptides were compared with the database to identify missed cleavage sites.

Peptide fingerprinting data analysis was conducted using UNIFI (version 1.9.4, Waters) software. Carbamidomethyl (C), deamidated (NQ), oxidation (M), and acetylation (N-TERM) were considered as potential chemical modifications during database searching. The mass-to-charge ratio (*m*/*z*) tolerance was set to 20 ppm, and the fragment match tolerance was set to 20 ppm.

#### 3.2.2. Exclusion of Signature Peptides with Glycosylation Sites

The UniProt protein database was used to search for reports related to the glycosylation sites of IL-6. The DTU Health Tech 4.0 database was employed for the prediction of glycosylation sites, and only sites scoring above 0.5 were considered to be possibly glycosylated. Peptides with reported or predicted glycosylation sites were excluded.

#### 3.2.3. Exclusion of Signature Peptides with Disulfide Bond Sites

The UniProt protein database was used to search for reported disulfide bonds in the sequence of IL-6, and the peptides containing these sites were excluded.

### 3.3. Preparation of Stock Solutions and Working Solutions

In order to prepare the amino acid stock solutions of the reference materials and isotope-labeled internal standards, initial samples were dissolved in ultrapure water to a concentration of 1 μg/mg. In order to prepare the signature peptide stock solutions of the in-house reference materials and isotope-labeled internal standards, initial samples were dissolved in ultrapure water to a concentration of 1 μg/mg. The stock solutions were divided into aliquots and stored at −80 °C. Prior to use, the stock solutions were further diluted with ultrapure water to prepare the working solutions.

### 3.4. Quantification of IL-6 via IDMS Based on Amino Acid Assay

All sample preparations were carried out using the weighing method and were conducted as follows: 30 mg of the IL-6 sample and 100 mg of the mixed isotope-labeled amino acid working solution were added to a vial. To hydrolyze the IL-6 protein into its constituent amino acids, the sample was acid-digested with 6 mol/L of hydrochloric acid in an oven at 110 °C. Once hydrolyzed completely, the samples were dried and redissolved with 0.2% acetonitrile in water.

An HP-C18 column (3.0 μm, 2.1 × 150 mm, Sepax Technologies, Inc., Suzhou, China) was used for chromatographic separation. The samples were eluted with the flow rate set to 0.2 mL/min under a linear elution program with the mobile phase (0.8% formic acid and 2% acetonitrile in ultrapure water). The sample injection volume was 10 µL.

The mass detector was set to the positive ionization, multiple reaction monitoring (MRM) mode with an electrospray ionization (ESI) ion source. The ion source parameters were as follows: capillary voltage, 3500 V; cone voltage, 50 V; cone gas, 150 L/h; desolvation gas flow, 800 L/h; ion source temperature, 150 °C; and desolvation temperature, 350 °C. The quantification ion pairs used for MS detection were *m/z* 132.1 > *m/z* 86.0 for leucine, *m/z* 139.1 > *m/z* 92.0 for isotope-labeled leucine (^13^C_6_, ^15^N); *m/z* 116.1 > *m/z* 70.0 for proline, *m/z* 122.1 > *m/z* 75.0 for isotope-labeled proline (^13^C_5_, ^15^N); *m/z* 118.1 > *m/z* 72.0 for valine, *m/z* 124.1 > *m/z* 77.0 for isotope-labeled valine (^13^C_5_, ^15^N); and *m/z* 166.1 > *m/z* 120.0 for phenylalanine, *m/z* 176.1 > *m/z* 129.0 for isotope-labeled phenylalanine (^13^C_9_, ^15^N).

### 3.5. Quantification of IL-6 via IDMS Based on Signature Peptides

All the sample preparations were carried out using the weighing method and were conducted as follows: 30 mg of the IL-6 samples, 45 mg of mixed isotope-labeled signature peptide working solution, 20 μL of enzymatic digestion buffer (20 mM pH 8.0, 150 mM NaCl), 60 μL of acetonitrile, and 30 μL of trypsin (0.1 mg/mL) were added into a vial. Ultrapure water was added to bring the total volume of the mixed solution to 200 μL. The reaction was carried out in a constant temperature shaker at 37 °C, with trypsin added every 12 h. After the reaction, formic acid was added to the system to achieve a concentration of 2.5% to stop the reaction.

The samples were desalted and separated on C18 SPE columns (Sep-Pak, Waters, Milford, CT, USA). Next, 100% acetonitrile was used to activate the SPE column, and 0.1% formic acid was used to equilibrate the column. The sample was diluted with ultrapure water to achieve a formic acid mass fraction of approximately 1% before being loaded into the column and then washed with 1.5 column volumes of 0.1% formic acid and eluted in 80% acetonitrile. The eluate was concentrated to dryness in a vacuum centrifuge and redissolved in 600 μL of 0.1% formic acid aqueous solution for further use.

### 3.6. LC-MS Conditions for IDMS Analysis of IL-6

An HP-C18 column (3.0 μm, 2.1 × 150 mm, Sepax Technologies, Inc., Suzhou, China) was used for chromatographic separation. The samples were eluted with the flow rate set to 0.2 mL/min under a gradient elution program with the mobile phase (mobile phase A: 0.1% formic acid acetonitrile solution; mobile phase B: 0.1% formic acid water solution). The following elution gradients were used: 0–2 min, 14% A: 86% B; 7 min, 42% A: 58% B; 10–12 min, 90% A: 10% B; 13 min, 14% A: 86% B. The sample injection volume was 10 µL.

The mass detector was set to positive ionization, MRM mode with an ESI ion source. The ion source parameters were as follows: capillary voltage, 3500 V; cone voltage, 50 V; cone gas, 150 L/h; desolvation gas flow, 800 L/h; ion source temperature, 150 °C; and desolvation temperature, 350 °C. The quantification ion pairs used for MS detection were *m/z* 560.6 > *m/z* 844.6 for signature peptide T6, *m/z* 562.6 > *m/z* 848.6 for isotope-labeled peptide T6, *m/z* 663.6 > *m/z* 812.5 for signature peptide T9, *m/z* 667.1 > *m/z* 819.5 for isotope-labeled peptide T9, *m/z* 495.0 > *m/z* 776.5 for signature peptide T15, and *m/z* 498.5 > *m/z* 783.5 for isotope-labeled peptide T15.

### 3.7. Concentration Calculation of IL-6

The raw MS data were processed and analyzed, which allowed us to determine the calibrated concentration of amino acids and signature peptides in the sample. To further ascertain the concentration of IL-6 protein in the sample, Equations (1) and (2) were introduced.

(1) $$C_{IL-6(AA)}=\frac{C_{AA}}{{MW}_{AA}\times Z_{AA}}\times {MW}_{IL-6}$$

In Equation (1), $C_{IL-6(AA)}$ represents the concentration of IL-6 based on a specific amino acid, $C_{AA}$ represents the calibrated concentration of this amino acid within the sample, ${MW}_{AA}$ represents the molecular weight of this amino acid, $Z_{AA}$ represents the number of molecules of this amino acid per IL-6 molecule, and ${MW}_{IL-6}$ represents the molecular weight of IL-6.

(2) $$C_{IL-6(peptide)}=\frac{C_{peptide}}{{MW}_{peptide}\times Z_{peptide}}\times {MW}_{IL-6}$$

In Equation (2), $C_{IL-6(peptide)}$ represents the concentration of IL-6 based on a specific signature peptide, $C_{peptide}$ represents the calibrated concentration of this signature peptide within the sample, ${MW}_{peptide}$ represents the molecular weight of this signature peptide, $Z_{peptide}$ represents the number of molecules of this signature peptide per IL-6 molecule, and ${MW}_{IL-6}$ represents the molecular weight of IL-6.

### 3.8. Method Validation

#### 3.8.1. Linearity and Sensitivity

The calibration curve was constructed using the peak area ratio of the in-house signature peptide reference material and the isotope-labeled signature peptide as the *x*-axis, and the accurately weighed mass ratio as the *y*-axis. The signal-to-noise ratio was defined as the ratio of the MS signal response intensity to the noise level. Sensitivity detection included the low limit of detection and the low limit of quantification. The low limit of detection was determined as the concentration at a signal-to-noise ratio of 3:1, and the low limit of quantification was determined as the concentration at a signal-to-noise ratio of 10:1.

#### 3.8.2. Accuracy

Accuracy was performed by comparing the IL-6 quantification results via IDMS based on the amino acid analysis and signature peptide. The content of amino acids and signature peptides was first quantified as described before. The content of IL-6 was calculated by Equation (1) or Equation (2). Statistical analysis was conducted to compare whether there were significant differences between these two quantitative methods.

#### 3.8.3. Specificity

Specificity was evaluated by analyzing the trypsin lysates of HEK293 cells, human serum, and bovine serum albumin. The pretreatment and detection of the samples were carried out according to Section 3.5 and Section 3.6.

#### 3.8.4. Sample Matrix Effects

To study the sample matrix effects, we added purified IL-6 protein to human serum to simulate real samples. We prepared serum samples with high (21.62 μg/mL), medium (2.16 μg/mL), and low (0.22 μg/mL) IL-6 concentrations. The quantitative analysis of IL-6 protein in the samples was performed via IDMS based on signature peptides. The trypsin digestion process and LC-MS detection parameters refer to Section 3.5 and Section 3.6.

#### 3.8.5. Precision

Precision was verified at high (2.154 mg/mL), medium (0.215 mg/mL), and low (0.022 mg/mL) concentrations of IL-6 samples, with IL-6 diluted using the buffer (20 mM Tris pH 8.0, 150 mM NaCl). The pretreatment and detection of the samples were carried out according to the method outlined in Section 3.5 and Section 3.6. Six replicates of each concentration were prepared daily for three consecutive days, and the intra-day and inter-day precision for each concentration were calculated. The coefficient of variation (CV) was used to assess the degree of variation.

### 3.9. Statistical Analyses

A two-tailed Student’s *t*-test was used to compare the quantification results between different amino acids and the quantification results between the signature peptide-based IDMS and amino acid-based IDMS. One-way ANOVA was used to compare the quantification results of IL-6 based on different signature peptides. Statistical analysis was performed using IBM SPSS software (version 27.0 for Windows).

## 4. Conclusions

In the present study, an IDMS method for quantifying IL-6 based on signature peptides was developed. In this method, suitable signature peptides are screened by analyzing missed cleavage sites, glycosylation sites, etc. T6, T9, and T15 were ultimately selected as the signature peptides for the quantification of IL-6. For purified IL-6 protein, the quantification results of all three signature peptides showed no significant difference compared to amino acid-based IDMS, indicating the accuracy of this method. The correlation coefficient of the calibration curve of the three signature peptides exceeded 0.99 within the range of linearity and had appropriate detection limits. The analysis of different cell lysates and BSA indicated that the selected signature peptides were specific to the IL-6 protein. High, medium, and low IL-6 concentration samples were set for the matrix effect and precision testing. The results showed that this method has low matrix effects, and the coefficient of variation (CV) of the intra-day and inter-day tests for the samples were both less than 5%.

The purpose of this study was to develop a specific IDMS quantification method for IL-6, providing a reliable tool for clinical testing and reference materials development. Compared to immunoassays, which require both specific antibodies for detection and purified protein for calibration, the IDMS quantification method based on signature peptides developed in this work only requires the synthesized signature peptides and isotope-labeled peptides. The key advantage of this method is that peptides are much more convenient for storage and operation.

## Figures and Tables

**Table 1:** Results of the peptide fingerprinting of IL-6.

No.	Start	End	*m*/*z*	Mr (Observed)	Mr (Expected)	Charge States	Δ (ppm)	Peptide
T2	38	44	765.4112	764.4032	764.3922	([M+H]^+^)	14.3905	DVAAPHR
T3	45	52	459.2431	916.4702	916.4608	([M+2H]^2+^)	10.2568	QPLTSSER
T4	53	55	375.2339	374.2259	374.2203	([M+H]^+^)	15.0099	IDK
T5	56	58	416.2694	415.2614	415.2536	([M+H]^+^)	18.7837	QIR
T6	59	68	560.8292	1119.6424	1119.6282	([M+2H]^2+^)	12.6828	YILDGISALR
T7	70	74	651.2948	650.2868	650.29	([M+H]^+^)	−4.9209	ETCNK
T8	75	84	942.3922	941.3842	941.3789	([M+H]^+^)	5.6300	SNMCESSK
T9	83	94	663.3702	1324.7244	1324.698	([M+2H]^2+^)	19.9291	EALAENNLNLPK
T10	95	98	478.2424	477.2344	477.225	([M+H]^+^)	19.6972	MAEK
T11	99	114	945.9405	1889.8650	1889.8344	([M+2H]^2+^)	16.1919	DGCFQSGFNEETCLVK
T12	115	132	1107.5979	2213.1798	2213.1612	([M+2H]^2+^)	8.4043	IITGLLEFEVYLEYLQNR
T13	133	141	541.7477	1081.4794	1081.467	([M+2H]^2+^)	11.4659	FESSEEQAR
T14	142	148	764.4104	763.4024	763.3891	([M+H]^+^)	17.4223	AVQMSTK
T15	149	156	494.8335	987.6510	987.642	([M+2H]^2+^)	9.1126	VLIQFLQK
T16	160	178	993.5416	1985.0672	1985.031	([M+2H]^2+^)	18.2365	NLDAITTPDPTTNASLLTK
T17	179	196	1105.0891	2208.1622	2208.1466	([M+2H]^2+^)	7.0647	LQAQNQWLQDMTTHLILR
T18	197	199	381.2188	380.2108	380.2052	([M+H]^+^)	14.7289	SFK
T19	200	207	490.2754	978.5348	978.5416	([M+2H]^2+^)	−6.9491	EFLQSSLR
T20	208	210	359.2444	358.2364	358.2321	([M+H]^+^)	11.8750	ALR
T2 + T3	38	52	555.2987	1662.8721	1662.8431	([M+3H]^3+^)	17.4400	DVAAPH(R)QPLTSSER
K + T7	69	74	779.3931	778.3851	778.3850	([M+H]^+^)	0.1632	(K)ETCNK
T10 + T11	95	114	784.0383	2349.0909	2349.0495	([M+3H]^3+^)	17.6241	MAE(K) DGCFQSGFNEETCLVK
T17 + T18	179	199	857.7833	2570.3259	2570.3421	([M+3H]^3+^)	−6.3027	LQAQNQWLQDMTTLIL(R) SFK

**Table 2:** Mass fraction of signature peptides in in-house reference materials.

Peptide	Mass Fraction of Signature Peptides (mg/g)			Average (mg/g)	CV (%)
	Based on Leucine	Based on Proline	Based on Valine		
T6	717.43	/	/	717.43	1.45
T9	804.89	809.90	/	807.40	2.50
T15	874.87	/	842.84	858.86	3.63

**Table 3:** Calibration curves and low limits of signature peptides.

Sample	Linear Equation	Correlation Coefficient (r^2^)	Low Limit of Detection (ng/mL)	Low Limit of Quantification (ng/mL)
T6	y = 3.1498x − 0.4816	0.995	1.16	4.82
T9	y = 5.8697x − 0.0729	0.999	0.22	0.73
T15	y = 2.6930x − 0.3359	0.995	0.77	3.36

x represents the ratio of the MS detection peak areas of signature peptides to their isotope-labeled peptides, and y represents the concentration of signature peptides (ng/mL).

**Table 4:** Content of IL-6 based on different signature peptides (*n* = 3).

Sample	Based on T6		Based on T9		Based on T15	
	Content (mg/mL)	CV (%)	Content (mg/mL)	CV (%)	Content (mg/mL)	CV (%)
1	2.075	1.780	2.140	0.263	2.103	0.481
2	2.085	0.803	2.179	0.395	2.161	2.483
3	2.202	1.044	2.186	1.016	2.085	1.780
4	2.158	0.880	2.177	0.704	2.152	3.303
5	2.167	0.797	2.106	0.211	2.177	1.656
6	2.207	1.156	2.139	0.409	2.144	3.102
Average	2.149	2.653	2.155	1.447	2.137	2.517

**Table 5:** Accuracy of IL-6 quantification (*n* = 6).

Based on Signature Peptides			Based on Amino Acids		*p*-Value
	Average (mg/mL)	CV (%)	Average (mg/mL)	CV (%)	
T6	2.149	2.653	2.156	0.934	0.796
T9	2.155	1.447			0.970
T15	2.137	2.517			0.250

**Table 6:** Sample matrix effects detection results (*n* = 3).

Sample	High IL-6 Concentration		Middle IL-6 Concentration		Low IL-6 Concentration	
	Mean (μg/mL)	CV (%)	Mean (μg/mL)	CV (%)	Mean (μg/mL)	CV (%)
1	19.34	2.49	1.84	4.29	0.18	4.45
2	19.04	3.33	2.06	3.42	0.19	5.90
3	21.17	1.96	1.97	4.74	0.19	1.10
Average	19.85	5.81	1.96	5.65	0.19	3.09
Recovery	91.81%		90.74%		86.36%	

**Table 7:** Intra-day and inter-day precision results (*n* = 6).

		High IL-6 Concentration			Middle IL-6 Concentration			Low IL-6 Concentration		
		Mean (mg/mL)	CV (%)	Recovery (%)	Mean (mg/mL)	CV (%)	Recovery (%)	Mean (mg/mL)	CV (%)	Recovery (%)
Intra-day	Day 1	2.121	2.64	98.5	0.206	1.40	95.8	0.020	3.80	90.2
	Day 2	2.097	2.37	97.4	0.204	1.43	94.9	0.021	1.93	96.2
	Day 3	2.113	3.14	98.1	0.202	1.81	94.0	0.019	2.67	87.9
Inter-day		2.146	1.27	99.3	0.204	1.98	94.7	0.020	3.74	91.8

## Data Availability

The data supporting this study’s findings are available from the author, Zihong Ye, upon reasonable request.

## Supplementary Materials

The following supporting information can be downloaded at: https://www.mdpi.com/article/10.3390/ijms25126777/s1.
