# Supplementary material for: The Development of an Isotope Dilution Mass Spectrometry Method for Interleukin-6 Quantification
Source: Int J Mol Sci. 2024 Jun 20;25(12):6777. doi: 10.3390/ijms25126777 (PMC11203838; doi:10.3390/ijms25126777)
Supplement: Supplementary file 1 [file ijms-25-06777-s001.zip › ijms-3010087-supplementary.pdf]

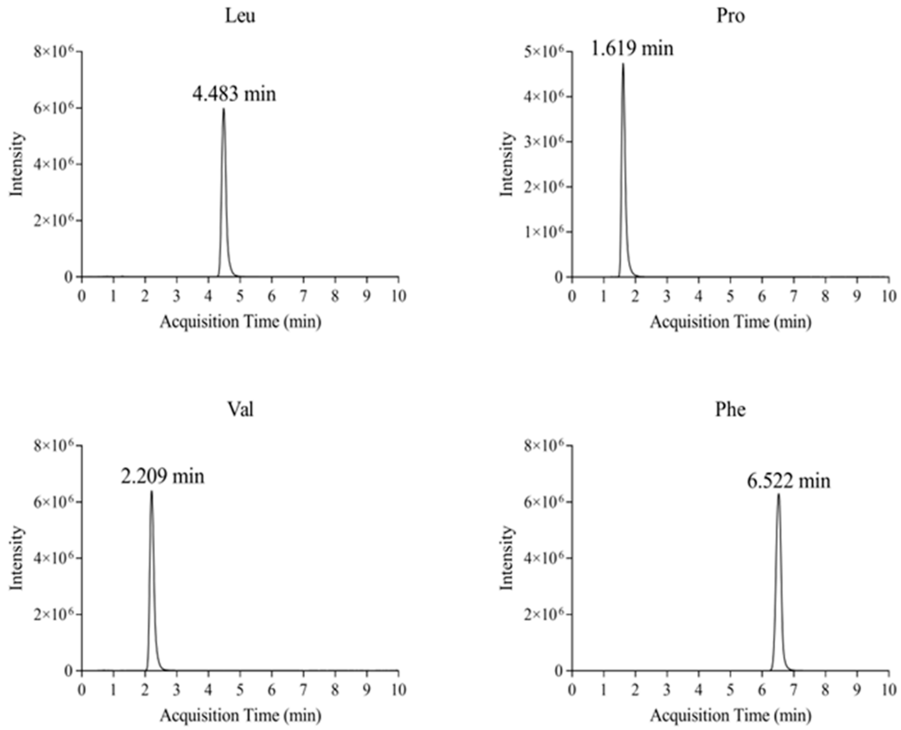

Supplementary Figure S1. Chromatograms of amino acid retention time. To achieve the chromatographic separation of amino acids, the chromatographic conditions were optimized. After optimizing the chromatographic conditions, we successfully separated leucine, proline, valine and phenylalanine on the chromatographic column, with retention times of 4.483 min, 1.619 min, 2.209 min, and 6.522 min, respectively.

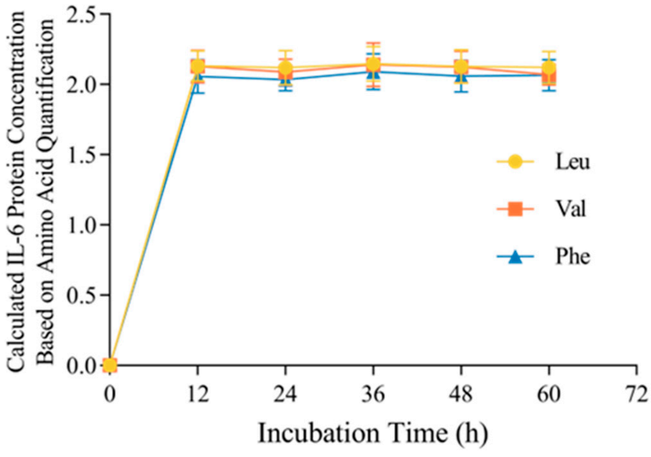

Supplementary Figure S2. Calculated IL-6 concentration after certain incubation time. To ensure the accuracy of our amino acid analysis results, we analyzed the relationship between the hydrolysis degree of IL-6 and incubation time. When the calculated IL-6 concentration no longer increased, we considered that IL-6 protein was 100% hydrolyzed.

In the present study, it was determined that the protein was fully hydrolyzed after 36 hours. Therefore, the optimum hydrolysis reaction time was set to 36 hours.
